# The Effect of 1 Year of Romosozumab on the Incidence of Clinical Vertebral Fractures in Postmenopausal Women With Osteoporosis: Results From the FRAME Study

**DOI:** 10.1002/jbm4.10211

**Published:** 2019-08-02

**Authors:** Piet Geusens, Mary Oates, Akimitsu Miyauchi, Jonathan D Adachi, Marise Lazaretti‐Castro, Peter R Ebeling, Carlos Augusto Perez Niño, Cassandra E Milmont, Andreas Grauer, Cesar Libanati

**Affiliations:** ^1^ Department of Internal Medicine, Rheumatology, Maastricht UMC, Maastricht, The Netherlands, and University Hasselt and ReumaClinic Genk Belgium; ^2^ Pacific Central Coast Health Center Santa Maria CA USA; ^3^ Miyauchi Medical Center Osaka Japan; ^4^ Department of Medicine, McMaster University Hamilton ON Canada; ^5^ Division of Endocrinology, Escola Paulista de Medicina, Universidade Federal de São Paulo São Paulo Brazil; ^6^ Department of Medicine School of Clinical Sciences, Monash University Clayton Australia; ^7^ UNIENDO Bogota Colombia; ^8^ Amgen Thousand Oaks CA USA; ^9^ UCB Pharma Brussels Belgium

**Keywords:** ANABOLICS, CLINICAL TRIALS, FRACTURE RISK ASSESSMENT, FRACTURE PREVENTION, OSTEOPOROSIS

## Abstract

Radiographic vertebral fractures (VFxs) are the most common fractures in osteoporosis and are associated with increased morbidity, mortality, and costs. A subset of VFxs manifest clinically, usually with a sudden onset of severe back pain. Romosozumab is a monoclonal antibody that binds and inhibits sclerostin, increasing bone formation and decreasing bone resorption, leading to rapid and large increases in bone density and strength and reduction in fracture risk. The FRAME (Fracture Study in Postmenopausal Women with Osteoporosis) study of postmenopausal women with osteoporosis demonstrated a significant reduction in new VFxs with romosozumab versus placebo. Here, we report the effect of romosozumab versus placebo on clinical VFx incidence over 12 months in women reporting back pain suggestive of VFxs. FRAME enrolled 7180 postmenopausal women with osteoporosis, mean age 70.9 years (hip *T*‐score −2.5 to −3.5). In the first year of the study, women received monthly romosozumab 210 mg (n = 3589) or placebo (n = 3591). At regular monthly visits, women reporting back pain suggestive of a clinical VFx had a confirmatory spine X‐ray. Clinical VFx risk in the romosozumab group versus the placebo group was calculated by Cox‐proportional hazards model. Of 119 women in FRAME with back pain suggestive of a clinical VFx over 12 months, 20 were confirmed to have experienced a new/worsening VFx. Three women receiving romosozumab had a clinical VFx (<0.1% of 3589 women) versus 17 (0.5% of 3591 women) receiving placebo resulting in a reduction in clinical VFx risk of 83% in the romosozumab group versus placebo through 12 months (HR 0.17; 95% CI, 0.05 to 0.58; *p* = 0.001). In the three romosozumab‐treated women, clinical VFxs occurred within the first 2 months of the study with no further clinical VFxs throughout the year. Romosozumab treatment for 12 months was associated with rapid and large reductions in clinical VFx risk versus placebo. © 2019 The Authors. *JBMR Plus* published by Wiley Periodicals, Inc. on behalf of American Society for Bone and Mineral Research.

## Introduction

Vertebral fractures (VFxs) are the most common fragility fractures and are the hallmark of osteoporosis, indicating a deficit in bone mass and microstructural deterioration.^1^ Importantly, they are associated with increased mortality, morbidity, and costs.^2^, ^3^ Both vertebral and hip fractures are associated with increased mortality; in fact, the increase in mortality following a VFx is similar or higher than that following a hip fracture.^4^, ^5^, ^6^, ^7^, ^8^ VFxs are associated with significant morbidity and are the only fragility fractures that result in permanent skeletal deformity leading to the development of the “dowager hump.” Although many are silent, around one‐third of these VFxs come to the attention of the patient and the clinician, usually because of the onset of severe back pain; they are referred to as clinical VFxs. Healthcare costs associated with clinical VFxs are high; the hospital costs of VFxs are second only to those of hip fractures.^9^

Romosozumab is a monoclonal antibody that binds and inhibits sclerostin.^10^ It exerts a dual effect on bone by increasing bone formation and decreasing bone resorption.^10^ The Fracture Study in Postmenopausal Women with Osteoporosis (FRAME) study compared the effect of romosozumab versus placebo on the cumulative incidences of new VFxs at 12 and 24 months in postmenopausal women with osteoporosis. In the FRAME study, romosozumab resulted in rapid and large BMD increases at the spine and hip^11^ in both the trabecular and cortical compartments.

Fragility fractures increase the risk of further fractures. Clinical VFxs in particular are associated with the highest recurrence of another fracture; rates as high as 25% have been observed over the subsequent year.^12^ Accordingly, therapies that rapidly reduce VFx risk are important for the management of patients with osteoporosis at high risk for fracture. Frequent monitoring of clinical VFxs in randomized clinical trials provides an opportunity to approximate the timing of the fracture, which is not possible with conventional radiographic evaluation of VFxs obtained at fixed intervals (ie, every 6 or 12 months). In the FRAME study, romosozumab resulted in a significant reduction in new VFxs and clinical fractures through 12 months compared with placebo. The relative risk reduction (RRR) on radiographic VFxs was 73% (*p* < 0.001).^11^ During the first year of the FRAME study, women attended monthly follow‐up visits allowing clinical VFxs to be identified in a timely manner.^11^ It was hypothesized that the early large gains in spine BMD observed during romosozumab therapy would correspond with an early decrease in risk of radiographic VFxs, as well as clinical VFxs. Therefore, in this prespecified exploratory study of FRAME, we analyzed the time‐to‐event for clinical VFxs in the romosozumab and placebo arms within 1 year.

## Subjects and Methods

### Study participants

A total of 7180 postmenopausal women were enrolled in the FRAME study (FRAME ClinicalTrials.gov number NCT01575834). The key inclusion criterion was a BMD *T*‐score <−2.5 at the total hip or femoral neck. Exclusion criteria included a history of hip fracture, any severe or more than two moderate VFxs, metabolic bone disease, and a BMD *T*‐score <−3.5 at the total hip or femoral neck (for full details, see reference 11).

### Study design

The details and primary results of the FRAME study have been published previously.^11^ The study was carried out in accordance with the World Medical Association Declaration of Helsinki—Ethical Principles for Medical Research Involving Human Subjects. Approval was provided by the ethics committee or institutional review board at each study center. All patients provided written informed consent. FRAME was an international, randomized, double‐blind, placebo‐controlled, parallel‐group trial. Randomization was stratified by age (<75 years, ≥75 years) and prevalent VFxs (yes, no). Women were randomized to receive s.c. romosozumab 210 mg per month or placebo for 12 months, followed by open‐label s.c. denosumab (60 mg every 6 months; Prolia, Amgen, Thousand Oaks, CA, USA) in both treatment arms for 12 months.^11^ All women received calcium (500 to 1000 mg) and vitamin D3 or D2 (600 to 800 IU) supplementation for the duration of the study.

### Identification of clinical VFxs

During the first 12 months of the FRAME study, a history and physical assessment were performed at each monthly visit to determine whether women had new back pain or worsening in previous back pain that was consistent with a possible VFx. A physical examination of the spine in the location of the pain with the presence of at least one of the following signs warranted further assessment: pain on closed fist percussion, palpable abnormality of the spine, and visible abnormality. If the results of the physical examinations were suggestive of a VFx, a lateral spine X‐ray was obtained for confirmation of new or worsening VFxs using the Genant method of semiquantitative grading to determine a change in vertebral deformity.^13^ Clinical VFx assessment was performed by a central imaging provider and blinded to treatment allocation.

### Data analysis

Analyses were primarily descriptive. The cumulative incidence of clinical VFxs was calculated in the romosozumab and placebo treatment arms. The HRs of clinical VFxs comparing romosozumab with placebo arms were evaluated using a Cox‐proportional hazards model stratified by age and prevalent VFx stratification factors.

### Data sharing

Qualified researchers may request data from Amgen clinical studies. Complete details are available at: http://www.amgen.com/datasharing

## Results

### Incidence of clinical VFxs

**Figure 1:**
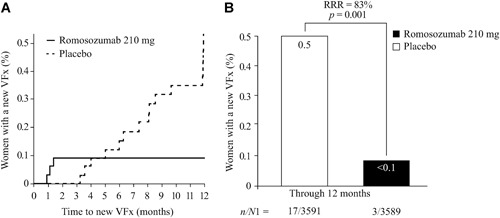
*(A)* Time to first new or worsening clinical VFx through month 12. (*B*) New or worsening clinical VFx incidence through month 12. RRR in clinical VFx for romosozumab 210 mg per month versus placebo = 83% (HR 0.17; 95% CI, 0.05 to 0.58; p = 0.001). RRR and *p* values were based on a Cox‐proportional hazards model adjusting for age and prevalent VFx stratification variables. *n/N*1*,* number of women with VFx/number of women; RRR = relative risk reduction; VFx = vertebral fracture.

Clinical VFxs were suspected in 119 women (47 with romosozumab, 72 with placebo) based on symptoms of new or worsening back pain and physical examination**.** In these 119 women, 20 were confirmed to have suffered a new (n = 19) or worsening (n = 1) VFx. Three of those VFx (two new, one worsening) occurred in the romosozumab group, all occurring within the first 2 months of study, with no additional clinical VFxs thereafter (Fig. 1 *A*). In the placebo group, 17 new clinical VFxs occurred through 12 months; RRR for romosozumab versus placebo was 83%; (HR 0.17; 95% CI, 0.05 to 0.58; p = 0.001; Fig. 1 *B*).

### Patient characteristics

In the romosozumab group, only 6.4% (3/47) of women with clinically suggestive back pain had actually developed a VFx; the proportion in the placebo group was higher at 23.6% (17/72). The baseline characteristics of the 119 women suspected of a possible VFx and referred for spine X‐ray are shown in Table 1. Women with a clinical VFx had a lower lumbar spine BMD *T*‐score (mean −3.25, romosozumab, −3.17, placebo) versus women without clinical VFx (mean −2.84, romosozumab, −2.86, placebo; Table 1). Risk values determined by the fracture risk assessment tool were similar in those with and without a new clinical VFx. All of the clinical VFxs in the romosozumab group, and the majority (82%) of the clinical VFxs in the placebo group, were moderate or severe (grade 2 or 3; Table 2).

**Table 1:** Baseline Characteristics of Women With New Back Pain Suggestive of a Possible Clinical VFx

Characteristic	No clinical VFx		Clinical VFx	
	Romosozumab 210 mg QM	Placebo	Romosozumab 210 mg QM	Placebo
	*N* = 44	*N* = 55	*N* = 3	*N* = 17
Age, mean (SD), years	70.2 (6.7)	71.1 (7.2)	73.3 (4.5)	72.1 (7.4)
BMD LS *T*‐score, mean (SD)	−2.84 (1.0)	−2.86 (1.0)	−3.25 (0.6)	−3.17 (1.1)
BMD total hip *T*‐score, mean (SD)	−2.62 (0.4)	−2.59 (0.4)	−1.75 (0.6)	−2.58 (0.5)
BMD femoral neck *T*‐score, mean (SD)	−2.74 (0.32)	−2.78 (0.32)	−2.62 (0.10)	−2.77 (0.32)
Prevalent VFx, *n* (%)	10 (22.7)	12 (21.8)	2 (66.7)	3 (17.7)
Prior non‐VFx at or after age 55 years, *n* (%)	11 (25.0)	17 (30.9)	1 (33.3)	6 (35.3)
FRAX score (10‐year probability of major osteoporotic fracture), median (range)	11.0	13.6	10.3	16.5
	(4.4 to 29.4)	(4.4 to 48.9)	(7.7 to 15.4)	(4.5 to 46.2)
FRAX score (10‐year probability of hip fracture), median (range)	4.4	6.5	3.7	7.7
	(1.6 to 12.0)	(1.4 to 34.2)	(2.9 to 5.1)	(1.5 to 35.6)

LS = lumbar spine; QM = per month; VFx = vertebral fracture; FRAX = fracture risk assessment tool.

**Table 2:** Grade of New Clinical VFx in the Romosozumab‐ and Placebo‐Treated Groups

	Clinical VFx	
	Romosozumab 210 mg QM	Placebo
Grade^a^	*N* = 3	*N* = 17
1	0	3 (17.65)
2	2 (66.67)	7 (41.18)
3	1 (33.33)	7 (41.18)

Values shown are *n* (%).

QM = monthly; VFx = vertebral fracture.

^a^ Grade of clinical VFx was assessed according to the method of Genant.^13^

## Discussion

In this post hoc analysis of the FRAME study, 12 months of romosozumab treatment led to a significant reduction in the incidence of clinical VFxs, which in the romosozumab group were only observed during the first 2 months of treatment, with no additional clinical VFx for the remainder of the 12‐month treatment period. The relevance of the significant reduction in clinical VFxs in the romosozumab group compared with the placebo group is compounded by the fact that the majority of confirmed clinical VFxs were moderate or severe. Moderate or severe vertebral fractures carry a greater risk of subsequent fractures than mild ones.^14^

These data complement the primary outcomes reported in the FRAME study where spine X‐rays obtained per protocol in all women at 6 and 12 months demonstrated an early effect in reducing morphometric VFxs with romosozumab. Indeed, there were 16 women with such fractures in the romosozumab group in the first 12 months, with 14 of them observed at month 6; only two additional women experienced fractures between months 6 and 12. In contrast, in the placebo group, VFxs were observed in 26 women by month 6; 33 additional women with VFxs were observed between months 6 and 12.^11^

New or worsening clinical VFxs occurred in the placebo group despite this group receiving calcium and vitamin D and generally maintaining BMD during the 12‐month study period. This underscores the importance of clinically relevant improvements in BMD to reduce fracture risk such as those resulting from romosozumab administration. BMD is an important determinant of fracture risk and increases in spine BMD with romosozumab are associated with a corresponding improvement in estimated bone strength.^15^ At the spine, romosozumab has demonstrated improvements in vertebral trabecular and cortical bone^15^ and significant increases in vertebral strength over 12 months compared with teriparatide and placebo.^16^

The identification of VFxs in clinical practice remains a challenge. In the current study, clinical evaluation had poor specificity to identify those with a true event, even in the hands of specialists and doctors knowledgeable about osteoporosis. Accordingly, our study emphasizes the need to obtain vertebral radiographs to ensure the accurate identification of VFxs, if back pain suggestive of the development of a VFx is present. In the current analysis of FRAME, there were 119 instances of such back pain, but a VFx was confirmed in 20 cases. Back pain, in patients known to have osteoporosis, merits investigation and imaging of the spine, as the diagnosis of a new VFx could result in changes in treatment to prevent further fractures in the near future.

It is well known that VFxs substantially increase the risk of subsequent fractures,^12^, ^17^, ^18^ and lead to increased morbidity and mortality,^3^, ^5^ as well as high costs.^9^ The incidence of another fragility fracture within the year following a clinical VFx has been recently shown to be approximately 16%.^19^ This extremely high risk mandates that these fractures be rapidly identified so that appropriate therapies be commenced without delay. This matter has been the subject of several recommendations including a recent call‐to‐action endorsed by multiple international and national societies.^20^

Unfortunately, most VFxs, whether clinical or radiographic, remain undiagnosed and untreated,^2^, ^21^ even after imaging of the spine is performed. Our data support the concept of performing a radiological evaluation of the spine in patients at risk, especially in those presenting with symptoms paying special attention to report and act upon vertebral fracture identification.

Our study has several limitations. The endpoint of new or worsening clinical VFxs was exploratory even though prespecified; despite the large cohort studied, there were relatively few clinical VFxs; the data may not be generalizable to men; and the limited number of events prevented robust characterization of the women or of the relationship between BMD and fracture risk reduction. Strengths of this study include its robust design to allow identification of clinical VFxs, the double‐blind nature of the assessments, and the consistency of the results.

In women receiving romosozumab, new or worsening clinical VFxs occurred only within the first 2 months of therapy with no further new clinical VFxs through the first 12 months. In conclusion, romosozumab therapy for 12 months was associated with rapid and large reductions in clinical VFx risk compared with placebo.

## Disclosures

PG has received grant/research support from Amgen, Pfizer, MSD, UCS, Abbott, Eli Lilly, BMS, Novartis, Roche, and Will‐Pharma, and participated in a speakers’ bureau/acted as a consultant for Amgen. MO has received grant/research support from Amgen, and participated in a speakers’ bureau/acted as a consultant for Amgen, Eli Lilly, and Radius, and as a speaker for Medtronic. AM has acted as a consultant for Amgen and Astellas BioPharma K.K. JDA has received grant/research support from Amgen, Eli Lilly, Merck, and worked on speakers’ bureau/acted as a consultant for Amgen and Merck. ML‐C has received grant/research support from Amgen, acted as a consultant for Amgen, Eli Lilly, and Sanofi, and participated in a speakers’ bureau for Sanofi. PRE has received grant/research support from Amgen, Novartis, and Eli Lilly, and acted as a consultant for Amgen, Eli Lilly, Gilhead, and Alexion. CAPN is employed by, and owns stocks in UNIENDO and Clinical Research. CEM is employed by, and owns stocks in Amgen. AG was employed by, and owns stocks in Amgen. CL is employed by, and owns stocks in UCB Pharma.
